# ICU staffing models and patient outcomes

**DOI:** 10.1186/s40560-019-0380-6

**Published:** 2019-04-24

**Authors:** Nobuaki Shime

**Affiliations:** Department of Emergency and Critical Care Medicine, Graduate School of Biomedical and Health Sciences, Hiroshima University, 1-2-3 Kasumi, Minami-ku, Hiroshima, 734-8551 Japan

**Keywords:** Intensive care unit, Structure, Closed, Intensivist

## Abstract

The study by Ogura et al. investigated the association between the structures of intensive care units (ICUs) affecting patient outcomes. A major limitation of this study is that the types of ICUs had not clearly been defined, and the provided definition had been made subjectively. Making an additional questionnaire or site-visits to completely define the types and to clarify the time-coverage is suggested. It would also be worthwhile to analyze whether the existence and density of “certified intensivists” and their involvement contribute to the outcome to determine whether physician quality affects critically ill patient care.

To the Editor,

I read with considerable interest the article published in a recent issue of *Journal of Intensive Care* by Ogura and colleagues [1], who have shown a significant association between the type of ICUs and outcomes of patients diagnosed with sepsis. They made a post hoc analysis using fairly large-scaled registry data of > 2400 cases from the Japan Septic Disseminated Intravascular Coagulation study and found that the length of intensive care unit (ICU) stay was shorter and mortality was lower for patients managed in “closed” ICUs. The study should be admired for shedding light on how the structure of ICUs affect patient outcomes, which is scarcely investigated outside North America [2–4].

As the authors described, however, a major limitation of their study is associated with its definition of types of ICUs, which was not clearly defined, and with the provided definition having been made subjectively. Given the nature of the study (post hoc analysis) and with merely 35 open- or closed-ICUs participating, it would not be impossible to redefine the structures, possibly by making an additional questionnaire or site-visits. By doing that, not only the consultation density but also time-coverage (24 h a day or not) could be evaluated [2]. This might also help to ensure non-exclusion of the data (~ 500 cases were excluded in their study) when ICUs are not clearly classified as closed or open, making the analysis more robust.

Ideally, fundamental definitions regarding ICU structures should be established by any professional intensive care society, not by individual researchers, for common understanding. This is a prerequisite for the professional academic society for further advancement.

In addition, the authors defined the type of ICUs by the degree of consultation (none or elective vs. mandatory) to intensivists. It is important to describe and assess who the “intensivists” were. To date, the number of certified intensivists by the Japanese Society of Intensive Care Medicine reaches barely ~ 1500. It would be worthwhile to analyze whether the existence and density of “certified intensivists” and their involvement in patient care, including sepsis management, really contributes to significant outcomes [3]. Furthermore, it would be of great interest to see whether the quality of physicians could affect critically-ill patient care [4].

I believe that clarification of these issues would be helpful for better understanding of the benefit of ICU models in sepsis management.

## Abbreviation

ICU: Intensive care unit

## References

[1] Ogura T, Nakamura Y, Takahashi K, Nishida K, Kobashi D, Matsui S. Treatment of patients with sepsis in a closed intensive care unit is associated with improved survival: a nationwide observational study in Japan. J Intensive Care. 2018; Sep 3;6:57. 10.1186/s40560-018-0322-8 PMC6122219 30202529

[2] Nishisaki A, Pines JM, Lin R, Helfaer MA, Berg RA, Tenhave T (2012). The impact of 24-hr, in-hospital pediatric critical care attending physician presence on process of care and patient outcomes. Crit Care Med.

[3] Petitti D, Bennett V, Chao Hu CK. Association of changes in the use of board-certified critical care intensivists with mortality outcomes for trauma patients at a well-established level I urban trauma center. J Trauma Manag Outcomes. 2012(Mar 6;6):3. 10.1186/1752-2897-6-3 PMC3313879 22394687

[4] Kerlin MP, Epstein A, Kahn JM, Iwashyna TJ, Asch DA, Harhay MO (2018). Physician-level variation in outcomes of mechanically ventilated patients. Ann Am Thorac Soc.

